# *Helicobacter pylori* Infection-Induced Hepatoma-Derived Growth Factor Regulates the Differentiation of Human Mesenchymal Stem Cells to Myofibroblast-Like Cells

**DOI:** 10.3390/cancers10120479

**Published:** 2018-11-30

**Authors:** Chung-Jung Liu, Yao-Kuang Wang, Fu-Chen Kuo, Wen-Hung Hsu, Fang-Jung Yu, Shuchen Hsieh, Ming-Hong Tai, Deng-Chyang Wu, Chao-Hung Kuo

**Affiliations:** 1 Division of Gastroenterology, Department of Internal Medicine, Kaohsiung Medical University Hospital, 100 Tz-You 1st road, Kaohsiung 807, Taiwan; pinkporkkimo@yahoo.com.tw (C.-J.L.); fedwang@gmail.com (Y.-K.W.); s339238@ms15.hinet.net (W.-H.H.); yufj@kmu.edu.tw (F.-J.Y.); dechwu@yahoo.com (D.-C.W.); 2 Center for Stem Cell Research, Kaohsiung Medical University, 100 Shih-Chuan 1st road, Kaohsiung 807, Taiwan; shsieh@faculty.nsysu.edu.tw (S.H.); mhtai@faculty.nsysu.edu.tw (M.-H.T.); 3 Department of Internal Medicine, Kaohsiung Municipal Hsiao-Kang Hospital, 482 Shanming Road, Kaohsiung 812, Taiwan; 4 School of Medicine, College of Medicine, E-Da Hospital, I-Shou University, 1 Yida Road, Yanchao District, Kaohsiung City 824, Taiwan; ed100418@edah.org.tw; 5 Department of Medicine, Faculty of Medicine, College of Medicine, Kaohsiung Medical University, 100 Shih-Chuan 1st Road, Kaohsiung 807, Taiwan; 6 Department of Chemistry, National Sun Yat-sen University, 70 Lienhai Rd., Kaohsiung 804, Taiwan; 7 Institute of Biomedical Science, National Sun Yat-sen University, 70 Lienhai Rd., Kaohsiung 804, Taiwan

**Keywords:** *Helicobacter pylori*, hepatoma-derived growth factor, mesenchymal stem cells, carcinoma-associated fibroblast, myofibroblast

## Abstract

Hepatoma-derived growth factor (HDGF) plays a critical role in tumor cell proliferation, anti-apoptosis, VEGF expression, lymph node metastasis and poor prognosis in human gastric cancer. Gastric cancer, as one of the most prevalent cancers worldwide, is the second leading cause of cancer-related mortality in the world for the prognosis of gastric cancer is generally poor, especially in patients with advanced stage. *Helicobacter pylori* (*H. pylori*) infection causes the chronic inflammation of stomach as well as the development of gastric cancer, with a three to six-fold increased risk of gastric cancer. Carcinoma-associated fibroblasts (CAFs) are myofibroblasts in tumor microenvironment, which possess various abilities to promote the progression of cancer by stimulating neoangiogenesis, proliferation, migration, invasion and therapy resistance of tumor cell. Mesenchymal stem cells (MSCs) are reported to promote tumor malignance through differentiation of MSCs toward CAFs. In the present study, we demonstrated that *H. pylori* infection promotes HDGF expression in human gastric cancer cells. HBMMSCs treated with HDGF assume properties of CAF-like myofibroblastic phenotypes, including expression of myofibroblast markers (α-smooth muscle actin (α-SMA), procollagen α1, tropomyoson I, desmin, fibroblast activation protein (FAP)), and fibroblast markers (prolyl-4-hydroxylase A1 (PHA1) and fibroblast specific protein-1 (FSP-1)/S100A4). HDGF recruits HBMMSCs, and then HBMMSCs further contributes to cell survival and invasive motility in human gastric cancer cells. Treatment of HDGF neutralizing antibody (HDGF-NAb) and serum significantly inhibit HDGF-regulated differentiation and recruitment of HBMMSCs. These findings suggest that HDGF might play a critical role in gastric cancer progress through stimulation of HBMMSCs differentiation to myofibroblast-like cells.

## 1. Introduction

Accumulating studies suggest that carcinoma-associated fibroblasts (CAFs) are involved in tumor development. Myofibroblasts in the tumor microenvironment (also called carcinoma-associated fibroblasts (CAFs) or cancer stroma) possess abilities to promote primary tumor growth and progression by inducing the processes of neoangiogenesis, tumor cell proliferation, survival, migration, invasion and therapy resistance [1,2]. CAFs isolated from gastric cancer may promote angiogenesis through high expression of galectin-1 [3]. CAFs contribute to gastric cancer cell invasion and peritoneal dissemination through epigenetic modulation and miR-200b repression [4]. FGF9 secreted from CAFs may be a possible mediator that promotes anti-apoptosis in gastric cancer cells [5]. Thus, CAFs might act as a therapeutic target for gastric cancer progression [6]. 

Mesenchymal stem cells (MSCs) possess the capacity of self-renewal, long-term viability, and differentiation potential toward various cell types, for example, adipogenic, osteogenic, and chondrogenic lineages [7]. Potential clinical applications of MSCs might contribute to the regenerative medicine. However, MSCs is reported to may be involved in adverse effect that leads to tumor growth. MSCs act as the important population of cells contributing to cancer progression and drug resistance [8,9]. MSCs might promote tumor malignance such as tumor growth, invasion, metastasis and therapy resistance through migrating to the damaged tissue and secreting many kinds of cytokines, chemokines and growth factors in the microenvironment of developing gastric tumor [10]. Furthermore, MSCs differentiate into CAFs that then strongly construct the tumor microenvironment [8,11,12,13,14,15,16]. So far, gastric cancer is one of the most prevalent cancers worldwide and is the secondary leading cause of cancer-related mortality [17] because of the poor prognosis of patients with advanced gastric cancer. Invasion and metastasis are the major causes of death from human gastric cancer. 

HDGF is a protein of 240 amino-acid that composed of a highly conserved N-terminal 100 residues HATH (homologous to the amino terminus of HDGF) domain and a variable C-terminal 140 residues (C140) domain. HDGF is originally purified from conditioned medium of HuH-7 liver cancer cells [18]. HDGF expression is reported to positively correlate with lymph node metastasis and prognosis of gastric cancer, acts as a prognostic factor for gastric cancer [19]. HDGF is also involved in cell proliferation, anti-apoptosis and VEGF expression in human gastric cancer [20,21]. The above findings suggest that HDGF might be a therapeutic target for gastric cancer.

In the present study, we investigated the effect of *H. pylori* infection on HDGF expression, the effect of HDGF on the differentiation of HBMMSCs toward CAFs, the recruitment of HBMMSCs by HDGF, and further observed if the capacity of human gastric cancer cell survival and invasive motility is upregulated by HBMMSCs. The results demonstrated that *H. pylori* infection induces HDGF expression, HDGF upregulates CAF markers in HBMMSCs, and recruits HBMMSCs; in which HBMMSCs promoted gastric cancer cell survival and invasive motility. 

## 2. Results

### 2.1. In Vitro Differentiation of Osteocytes, Adipocytes and Chondrocytes from Human Bone Marrow-Derived Mesenchymal Stem Cells

**Figure 1:**
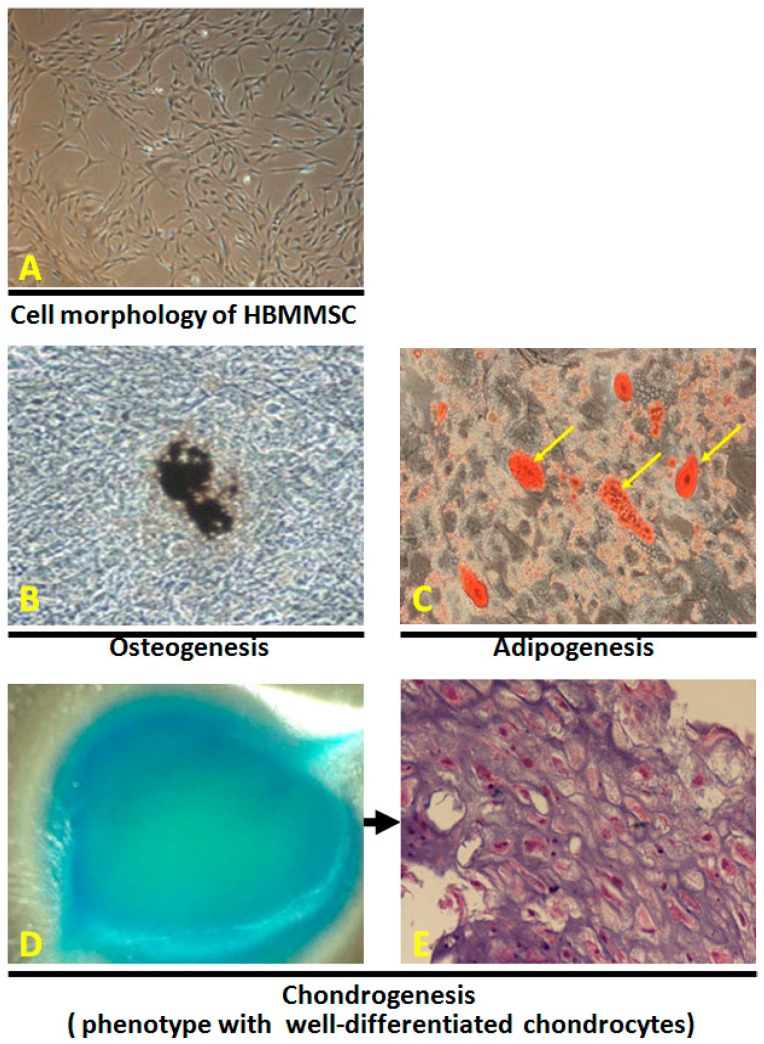
The morphology of HBMMSCs was showed by light microscopy (**A**). Osteogenic, chondrogenic, and adipogenic differentiation from HBMMSCs. Osteogenic differentiation from HBMMSCs was evidenced by cells morphology after 21 days of induction. Formation of mineralized matrix was shown by von Kossa staining (**B**, shown at original magnification ×200). Adipocytic differentiation from HBMMSCs was evidenced by the formation of lipid vacuoles by oil-red O staining (**C**, shown in phase-contrast photograph at original magnification ×200 and). Chondrogenic differentiation from HBMMSCs was evidenced by alcian blue staining (**D**), and by H&E staining for histological analysis (**E**).

To investigate the osteogenic potential of the human bone marrow-derived mesenchymal stem cells (HBMMSCs), P4 to P7 HBMMSCs (5 × 10^4^) (Figure 1A) were cultured in 6-well plate under conditions appropriate for inducing osteocyte differentiation. After 21 days of induction to differentiate under osteogenic conditions, the spindle shape of HBMMSCs flattened and broadened with increasing time of induction and formed mineralized matrix as evidenced by von Kossa staining (Figure 1B). To assess the adipogenic potential, P4 to P7 HBMMSCs (5 × 10^4^) were cultured in 6-well plate under adipogenic medium. After 21 days of induction, the change of cell morphology and the formation of neutral lipid vacuoles were noticeable and visualized by staining with oil-red O (Figure 1C). The chondrogenic potential of HBMMSCs (1 × 10^5^) was evaluated by culturing under the pelleted micromass system in chondrogenic medium. After 21 days of differentiation, cartilage was stained intense dark blue by alcian blue staining (Figure 1D). Chondrogenesis was confirmed by histological analysis for well-differentiated chontrocytes (Figure 1E). 

### 2.2. H. Pylori Infection Induces HDGF Expression in Human Gastric Cancer Cells

**Figure 2:**
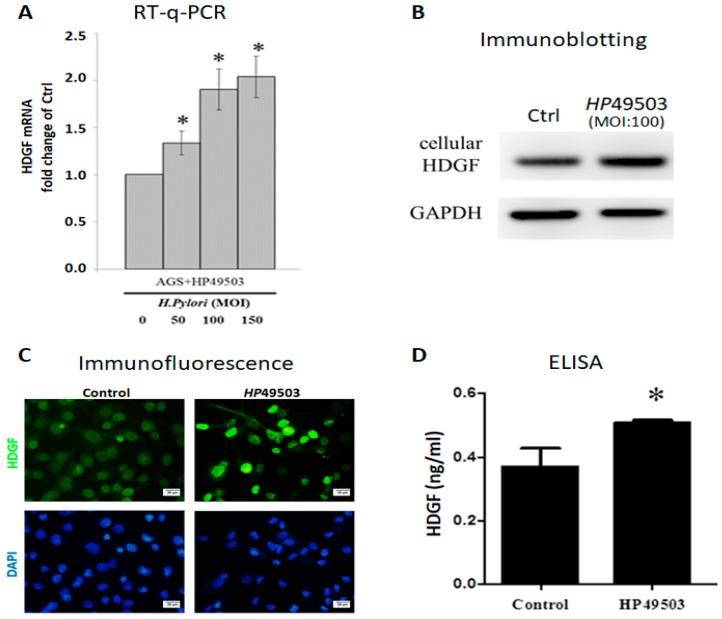
Human AGS cells with HP infection expresses high HDGF level. HDGF mRNA in AGS cells with HP49503 infection for 24 h was analyzed by RT-q-PCR (**A**). HDGF protein from AGS cells infected with HP49503 for 24 h was detected by immunoblotting assay. GAPDH was used as an internal control (**B**). Immunofluorescent staining was used to detect the expression of HDGF in AGS cells with HP49503 infection. Scale bar = 20 μm. (**C**). Secreted HDGF from AGS cells with HP49503 infection was measured by ELISA assay (**D**). * *p* < 0.05 versus control (mean ± SD, n = 3).

The effect of *H. pylori* infection on HDGF expression was analyzed in human gastric adenocarcinoma cells. HDGF protein expression was analyzed in the human gastric tissue with or without HP infection (HP-, *N* = 20 cases; HP+, *N* = 20 cases), using IHC staining. We found that HDGF is significantly expressed in HP-infected gastric tissues (Figure S1). However, we found serum HDGF is not significantly changed when compare HP-infected (HP+) patients before and after HP eradication (pre-TX and post-TX). We speculated that HP infection has induced the changes of many host’s genes and proteins by virulence factors of HP, even though HP is eradicated (Figure S2). We have established the HP49503 infection in the AGS cells culture. We observed the adhesion of HP49503 in the presence and absence of Clarithromycin (HP eradication), using immunefluorescence antibodies against HP and CagA. We found that HP49503 possesses the strong adhesion ability even though HP is eradicated (sterilized/killed) (Figure S3). Human AGS cells were infected with various MOI (0, 50, 100 and 150) of HP49503 for 24 h, and followed by RT-q-PCR analysis. HP49503 infection significantly induced HDGF mRNA expression (Figure 2A). A notable increase in HDGF protein level was observed in AGS cells with HP49503 infection, using immunoblotting and immunofluorescence staining assays (Figure 2B,C). Furthermore, the results from ELISA assay revealed that HP49503 infection could induce higher concentration of secreted HDGF form human AGS cells (Figure 2D).

### 2.3. HDGF Induces Expression of Myofibroblast and Fibroblast Markers in HBMMSCs 

**Figure 3:**
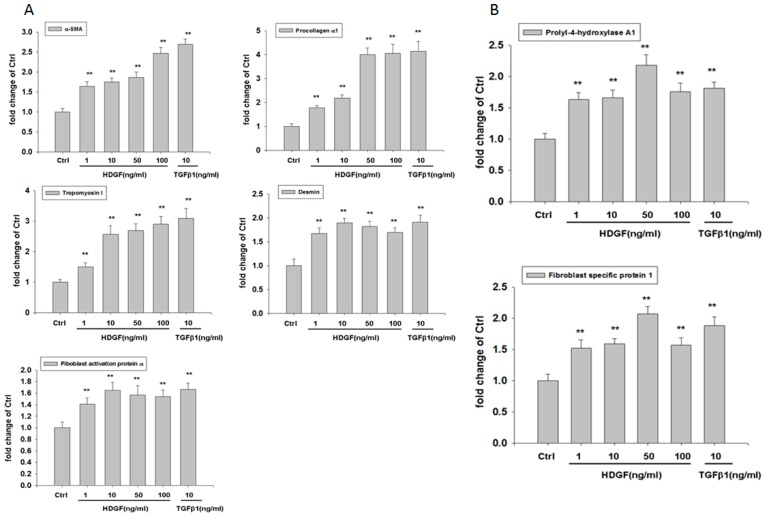
HDGF stimulates expression of myofibroblast (**A**) and fibroblast (**B**) markers in HBMMSCs in various concentrations. HBMMSCs were treated with HDGF (1, 10, 50, 100 ng/ml) for 24 hours. The effect of HDGF on marker expression of myofibroblast and fibroblast were measured by RT q-PCR. TGFβ1 (10 ng/ml) treatment as positive control. ** *p* < 0.01 versus control (mean ± SD, n = 3).

**Figure 4:**
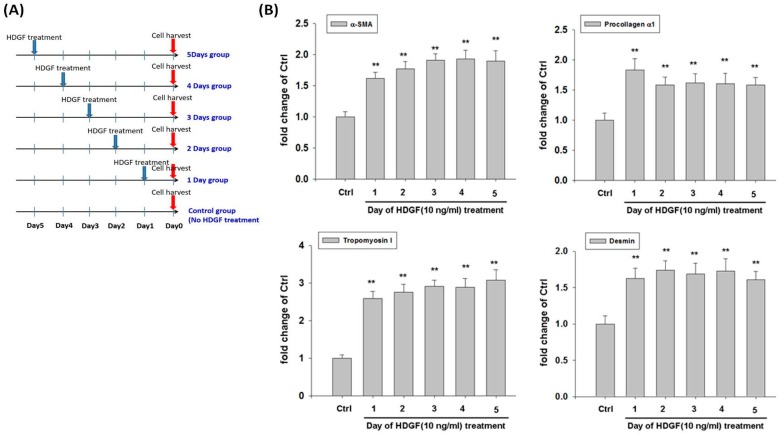
(**A**) A schematic representation showed the procedure of HDGF treatment in HBMMSCs culture. HBMMSC were treated with HDGF at various time points in the absence of serum. HBMMSC then were harvested at the same day. (**B**) HDGF stimulates expression of myofibroblast markers in HBMMSCs. HBMMSCs were treated with HDGF 10 ng/ml) for various periods (1, 2, 3, 4 or 5 days), respectively. The effect of HDGF on expression of myofibroblast markers was measured by RT q-PCR. ** *p* < 0.01 versus control (mean ± SD, n = 3).

To evaluate the influence of HDGF on expression of myofibroblast and fibroblast markers in HBMMSCs, we treated HBMMSCs with various concentration of HDGF (1, 10, 50, 100 ng/mL) or positive control TGFβ1 (10 ng/mL) for 24 h. The present study demonstrated HDGF increased myofibroblast markers, including α-smooth muscle actin (α-SMA), procollagen α1, tropomyoson I, desmin, fibroblast activation protein (FAP) (Figure 3A), and fibroblast markers, including prolyl-4-hydroxylase A1 (PHA1) and fibroblast specific protein-1(FSP-1)/S100A4 (Figure 3B). The effect of HDGF-upregulated myofibroblast marker was confirmed by treating HBMMSCs with HDGF (10 ng/mL) for various periods (1, 2, 3, 4, 5 days) (Figure 4). We also examined whether AGS cells could trigger the differentiation of HBMMSCs in the transwell co-culture system for 48 h. The results suggested that AGS cells might contribute to the differentiation of the HBMMSCs into myofibroblast-like cells (Figure S5).

### 2.4. HDGF Neutralizing Antibody and Serum Inhibits the Expression of Myofibroblast and Fibroblast Markers

**Figure 5:**
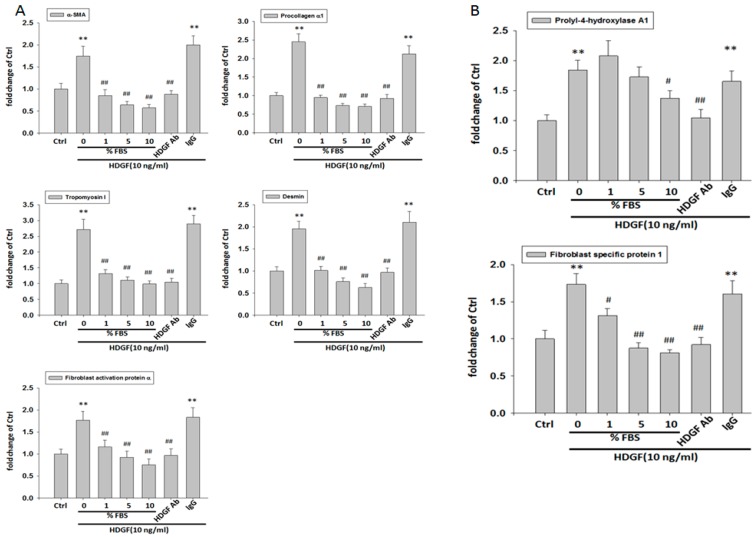
Serum and HDGF neutralizing antibody inhibits the expression of myofibroblast and fibroblast markers. HBMMSCs were treated with various concentration of FBS (0, 1, 5, 10%), or HDGF neutralizing antibody (HDGF-NAb; 1 μg/ml) in the presence of HDGF (10 ng/ml). After 24 hours, the marker expression of myofibroblast (**A**) and fibroblast (**B**) was measured by RT q-PCR. * *p* < 0.05; ** *p* < 0.01 versus control (line 1); # *p* < 0.05; ## *p* < 0.01 versus only HDGF treatment (line 2) (mean ± SD, n = 3).

We further analyzed the effect of HDGF neutralizing antibody (HDGF-NAb) and serum on HDGF-upregulated markers of myofibroblast and fibroblast in HBMMSCs. We found HDGF-induced overexpression of myofibroblast and fibroblast markers (α-smooth muscle actin (α-SMA), procollagen α1, tropomyoson I, desmin, fibroblast activation protein (FAP), prolyl-4-hydroxylase A1 (PHA1) and fibroblast specific protein-1 (FSP-1)/ S100A4 in HBMMSCs were significantly inhibited when using HDGF-Nab (1 μg/mL) and various concentration of serum (1%, 5%, 10%) in the culture system (Figure 5). These findings suggested that HDGF plays a critical role in inducing differentiation of HBMMSCs toward myofibroblasts and fibroblasts. 

### 2.5. HDGF Neutralizing Antibody Inhibits HDGF-Induced HBMMSCs Recruitment

**Figure 6:**
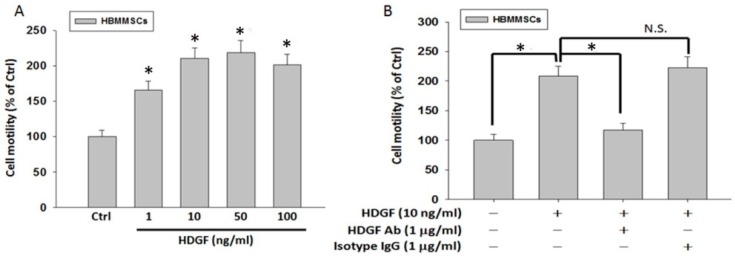
(**A**) HDGF induces HBMSCs motility at various concentrations. HBMMSCs were treated with HDGF (1, 10, 50, 100 ng/ml) for 24h. The effect of HDGF on motility capacity of HBMMSCs was measured by motility assay. (**B**) HDGF neutralizing antibody inhibits HDGF-mediated HBMMSCs recruitment. HBMMSCs treated with HDGF neutralizing antibody (HDGF-NAb; 1 μg/ml) in the presence or absence of HDGF (10 ng/ml) were analyzed for the inhibitory effect of HDGF-Nab on HDGF-mediated recruitment motility. * *p* < 0.01 versus control (line 1); * *p* < 0.01 versus only HDGF treatment (line 2) (mean ± SD, n = 3).

To identify the role of HDGF in the recruitment motility of HBMMSCs, we treated HBMMSCs with various concentrations (1, 10, 50 and 100 ng/mL) of recombinant HDGF for 24 h. Subsequently, the capacity of HBMMSCs recruitment was valued by cell motility assay (Figure 6A). 

We observed that HDGF in various concentrations (1, 10, 50 and 100 ng/mL) induce the significant increase in motility in HBMMSCs. However, the HDGF-induced HBMMSCs recruitment motility was significantly inhibited when using HDGF neutralizing antibody (HDGF-NAb) (1 μg/mL) in the culture system (Figure 6B). We have examined the chemotactic potential of AGS ± Hp in the presence or absence of HDGF. The results suggested that HP infection and/or HDGF treatment enhance the capacity of HBMMSCs recruitment (Figure S4). These findings demonstrated that HDGF has an important role in the induction of recruitment motility in HBMMSCs. 

### 2.6. HBMMSCs Upregulate Survival and Invasive Motility in Human Gastric Cancer Cells

**Figure 7:**
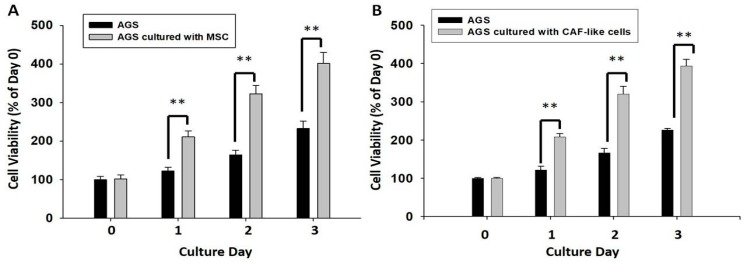
HBMMSCs (**A**) or CAF-like cells (**B**) enhance survival in human gastric cancer cells. HBMMSCs or CAF-like-cells were co-cultured with human AGS gastric cancer cells for various periods (1, 2, 3 days). The effect of HBMMSCs or CAF-like cells on cellular survival of human gastric cancer cells was measured. The responses to HBMMSCs or CAF-like cells were measured by MTT assay. ** *p* < 0.01 versus only AGS cells (mean ± SD, n = 3).

We further evaluated the effect of HBMMSCs on survival and invasive motility of human gastric cancer cells. To determine the effect of HBMMSCs on human gastric cancer cell proliferation, AGS cells (lower compartment) were co-cultured with HBMMSCs (upper chamber) for 24, 48, and 72 h and were subjected to MTT assay. HBMMSCs were pretreated with HDGF (1 ng/mL) for 24h for differentiating into CAF-like cells, and then co-cultured with human gastric cancer cells. We found that CAF-like cells contribute to the survival of human gastric cancer cells (Figure 7). 

**Figure 8:**
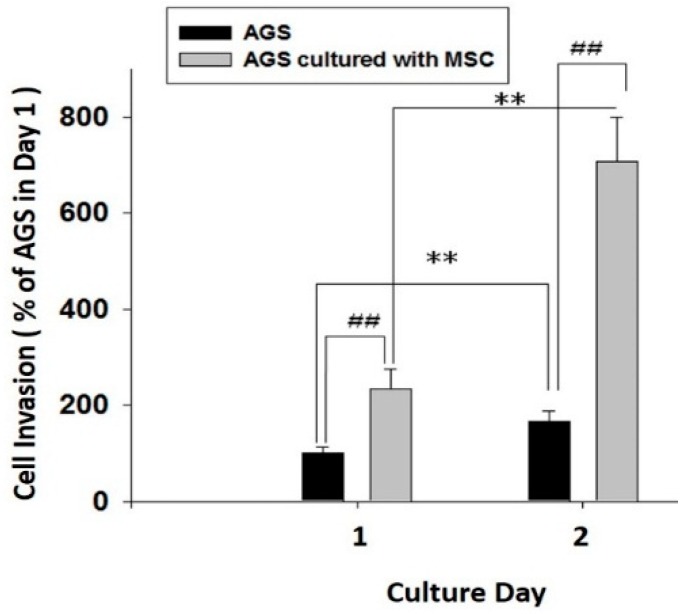
HBMMSCs enhances invasive motility in human gastric cancer cells. HBMMSCs and human AGS gastric cancer cells were cultured for 24 and 48 h, respectively. The effect of HBMMSCs on cellular motility of human gastric cancer cells were measured. The responses to HBMMSCs were measured by invasive motility assay. ** *p* < 0.01, only AGS cells in day 2 versus only AGS cells in day 1; ## *p* < 0.01, AGS cells cultured with MSC versus only AGS cells (mean ± SD, n = 3).

Human AGS gastric cancer cells were placed in the upper chamber pre-coated with collagen I; and lower compartment was seeded with HBMMSCs in the co-culture system. The co-culture system was filled with serum- and phenol red-free medium and incubated for 24 and 48 h, respectively. We found that HBMMSCs significantly contributed to invasive motility activity in human gastric cancer cells (Figure 8). The present result confirmed HBMMSCs significantly contribute to increase the capacity of survival and invasive motility in human gastric cancer cells.

## 3. Discussion

Major findings of this study can be summarized as the followings: (i) Results from multilineage differentiation showed that HBMMSCs remarkably possess the potential for osteogenic differentiation, adipogenic differentiation and chondrogenic differentiation. (ii) *H. pylori* infection induces HDGF expression in the human gastric tissue and human gastric cancer cells. (iii) Exposure to HDGF make HBMMSCs overexpress carcinoma-associated myofibroblast markers (α-smooth muscle actin (α-SMA), procollagen α1, tropomyoson I, desmin, fibroblast activation protein (FAP), and fibroblast markers (prolyl-4-hydroxylase A1 (PHA1) and fibroblast specific protein-1 (FSP-1)/S100A4). (iv) HDGF neutralizing antibody (HDGF-NAb) significantly inhibit HDGF-mediated expression of carcinoma-associated myofibroblast/fibroblast markers in HBMMSCs. (v) HDGF-NAb significantly inhibits HDGF-mediated HBMMSCs recruitment. (vi) HBMMSCs and HDGF-induced CAF-like cells significantly contribute to cell survival and invasive motility in human gastric cancer cells. 

Evidences suggest that CAFs play the critical role in the tumor growth and cancer progression. Interactions between CAFs, carcinoma cells and other tumor stroma cells are demonstrated to be crucial for aggressive tumor behaviors. CAF myofibroblasts are frequently detected in the tumor stroma part of various human cancers such as breast cancer, lung cancer, colon cancer and pancreas cancer et al. [22,23]. CAF myofibroblasts are reported to possess the abilities to upregulate tumor growth and progression through promotion of cell proliferation, migration, invasion and neoangiogenesis et al. Bone marrow-derived myofibroblasts have been shown to be one source of CAFs and promote tumor growth [24]. Cancer-associated fibroblasts activated by chemotherapy have been shown to maintain cancer-initiating cells (CICs) and their drug resistance through secretion of IL-17A [25]. To identify CAFs in cancer, the specific marker is necessary. The most widely used marker for CAFs is α-smooth muscle actin (α-SMA) and α-SMA has been known as a specific marker for myofibroblasts [26]. Fibroblast activation protein (FAP) [27], fibroblast specific protein-1 (FSP-1) /S100A4 [28] and prolyl 4-hydroxylase [29] are considered to be specific markers for CAF myofibroblasts. In the present study, we observed that HBMMSCs overexpress CAF myofibroblast markers (α-smooth muscle actin (α-SMA), procollagen α1, tropomyoson I, desmin, fibroblast activation protein (FAP), fibroblast markers (prolyl-4-hydroxylase A1 (PHA1) and fibroblast specific protein-1 (FSP-1)/S100A4) when exposed to HDGF in various concentrations and periods.

Mesenchymal stem cells (MSCs) have the differentiation potential toward diverse cell types, such as adipogenic, osteogenic, and chondrogenic lineages [7]. It suggests the potential applications of MSCs for clinical regenerative medicine. In addition, the capacity of MSCs migrating to and engrafting into the microenvironment of gastric tumor have attracted attentions [10]. MSCs have been show to upregulate tumor growth by migrating to the developing intrahepatic cholangiocarcinoma via SDF-1/CXCR4 signaling pathway, and MMP2 molecular factor in human medulloblastoma. MSCs then contribute to angiogenesis via VEGF, MCP-1 and HIF-1 signaling pathways [30,31]. HBMMSCs-secreted IL-8 upregulates the capacity of cellular invasive motility in human gastric cancer cells [14]. HBMMSCs contribute to breast tumor progression through becoming activated carcinoma-associated myofibroblasts [8,11,12,13]. In the present study, we observed HDGF treatment stimulates the differentiation of HBMMSCs toward CAF-myofibroblast. Treatment of HDGF-NAb on HBMMSCs confirms the effect of HDGF in controlling the differentiation of HBMMSCs toward CAF-myobroblasts. Furthermore, HBMSCs contribute to survival and invasive motility of human gastric cancer cells in the co-culture system. 

HDGF is closely involved in the disease progression of gastric cancer, which substantially affects the prognosis [19]. HDGF is highly expressed and functions as a mitogen that regulates proliferation in tissues such as the liver, kidney, lungs and blood vessels during embryonic development; however, HDGF level becomes notably lower after birth [32,33,34,35]. Clinical studies reveal that high HDGF levels also occur in non-small cell lung cancer, colorectal cancer, pancreatic cancer and melanoma. A large amount of the evidence suggests that HDGF overexpression is associated with the aggressive phenotypes of cancer cells, such as proliferation, invasiveness, and metastasis [36,37,38,39,40]. HDGF level shows a negative correlation with the survival rate of patients in the clinical studies. Therefore, HDGF is considered as an independent prognostic factor in patients with hepatocellular carcinoma [41], gastric cancer [19], pancreatic cancer [42], non–small-cell lung cancers [42], and gastrointestinal stromal tumors [43]. However, the mechanism behind HDGF-mediated gastric cancer development requires verification. In the present study, we found that HDGF significantly recruits HBMMSCs and stimulates the differentiation of HBMMSCs toward CAF-myofibroblasts, which further contributes to the progression of gastric cancer. 

The function of HDGF in cell survival and tumor formation has been extensively investigated. HDGF overexpression has been implicated in the development of malignant tumors; however, the mechanism behind HDGF regulation is largely unknown. In the present study, we observed that HDGF recruits HBMMSCs, and stimulates the differentiation of CAF-myofibroblasts from HBMMSCs. HBMMSCs further upregulate the capacity of survival and invasive motility in human gastric cancer cells. These findings suggest that HDGF might play a critical role in the progression of gastric cancer.

## 4. Materials and Methods 

### 4.1. Cells, Antibodies, Reagents and Enzymes

HBMMSCs (ATCC^®^ PCS-500-012™) were obtained from the American Tissue Culture Collection (ATCC, Manassas, VA, USA). Human AGS gastric cancer cells (ATCC^®^ Number: CRL-1739™) were obtained from the American Type Culture Collection/Bioresource Collection and Research Center (BCRC, Hsinchu, Taiwan). HDGF and HDGF neutralizing antibody were gift from Prof. Ming-Hong Tai. The recombinant HDGF proteins and anti-HDGF antibodies were generated as previously described [36]. The cDNA of human HDGF was amplified from a human fetal brain cDNA library (Stratagene, La Jolla, CA, USA) by polymerase chain reaction (PCR). Human HDGF cDNA were cloned by PCR primers that designed based on the HDGF sequence in the Gen-Bank database22 (accession number, NM_004494; forward primer, 5′-gccatgtcgcgatccaaccggca gaa-3′; reverse primer, 5′-ctacaggctctcatgatctctg-3′). The PCR-amplified HDGF cDNA was subcloned into the NdeI and XhoI cutting sites in the pET15b vector (Novagen, Madison, WI, USA), and then transformed into BL-21 cells (DE3, pLysS; Novagen). HDGF protein with 6×-histidine was purified using an NTA-agarose affinity column (Qiagen, Hilden, Germany). The generated recombinant protein was passed through Detoxi-Gel (Pierce Biotechnology, Rockford, IL, USA) to reduce contamination by endotoxin. HDGF antibodies were generated by periodic injection of recombinant HDGF protein into rabbits. The serum of immunized rabbits was collected and analyzed using immunoblotting.

### 4.2. Cell Culture

HBMMSCs (ATCC^®^ Number: PCS-500-012™) were obtained from the American Tissue Culture Collection. HBMMSCs were cultured in Iscove’s modified Dulbecco’s medium (IMDM, Gibco, Grand Island, NY, USA) and 10% fetal bovine serum (FBS, Hyclone, Logan, UT, USA) containing 10 ng/mL basic FGF-2 (Sigma Chemical Co., St. Louis, MO, USA), 5 ng/mL EGF (R&D Systems, Minneapolis, MN, USA), 100 units penicillin, 1000 units streptomycin, and 2 mM L-glutamine. 

Human AGS cells (ATCC^®^ Number: CRL-1739™) were cultured in RPMI-1640 medium containing 100 μg/mL penicillin, 100 μg/mL streptomycin, 2 mM glutamine, 1 mM HEPS buffer, and 10% fetal bovine serum (Life Technologies, Carlsbad, CA, USA). Cell culture was maintained at 37 °C in a humidified 5% CO_2_ atmosphere. 

### 4.3. H. Pylori Culture

*Helicobacter Pylori* strain ATCC 49503 (HP49503) was obtained from Bioresource Collection and Research Center (BCRC). HP49503 was cultured on BBLTM CDC Anaerobe 5% Sheep Blood Agar (BD Biosciences) at 37 °C in a microanaerobic chamber. HP49503 was collected and resuspended in cold phosphate buffered saline (PBS), and then used for AGS cells in the co-culture experiments.

### 4.4. Immunoblotting

For isolation of total proteins, cells were rinsed with cold PBS and resuspended in lysis buffer (0.5 M NaCl, 50 mM Tris, 10% glycerol, 1.0 mM EDTA, 1mM BME, 1% NP40, plus proteinase inhibitor cocktail and phosphatase inhibitor cocktail (Roche Molecular Biochemicals, Mannheim, Germany). The supernatant was collected by centrifugation at 12,000 g for 15 min at 4 °C. The concentration of isolated protein was determined by the Bradford method. Isolated proteins (40 μg) were loaded and analyzed by immunoblotting. Membranes with transferred protein were incubated with primary antibodies in the blocking buffer (5% non-fat dry milk, 20 mM Tris-HCl, pH 7.6, 150 mM NaCl, and 0.1% Tween 20 on an orbital shaker at 4 °C overnight. Membranes then were incubated with horseradish peroxidase-linked secondary antibodies (anti-rabbit, anti-mouse, or anti-goat IgG). Antibody-bound protein bands were detected using high sensitive Immoblot Western Chemiluminescent HRP Substrate (Millipore, Billerica, MA, USA), and photographed with Bio-Rad Chemiluminescence Imaging System (Bio-Rad Laboratories, Inc. Hercules, CA, USA)

### 4.5. Immunofluorescence Assay

AGS cells were seeded in 6-well plates (Corning Incorporated Life Sciences, Tewksbury, MA, USA). The next day, AGS cells were cultured with HP49503 for 24 h. Cells were rinsed with cold PBS, and fixed with 4% paraformaldehyde and permeabilized in Triton X-100. After washing with cold PBS, slides were blocked with 2% bovine serum albumin (BSA). Primary and secondary antibodies were incubated with 5% BSA in PBS. DAPI reagent was used as a mounting and counterstaining media.

### 4.6. Immunohistochemical Staining

Immunohistochemistry for HDGF was done on formalin-fixed, paraffin embedded tissues cut into serial 4-μm sections. After deparaffinization and rehydration, tissue sections for staining of HDGF were pretreated by microwave twice for 5 min in Dako REAL Target Retrieval Solution (Dako, Santa Clara, CA, USA). HDGF was detected with a rabbit antihuman polyclonal antibody (1:200; R&D Systems). The primary antibodies were applied to the slides and incubated overnight in humidified boxes at 4 °C. After incubation for 1 h at room temperature with peroxidase-conjugated corresponding secondary antibody, slides were counterstained with hematoxylin for visualization of the nucleus.

### 4.7. ELISA

The mouse anti-HDGF monoclonal antibody against C140 domain (C-terminal) was used as the detection antibody. Recombinant HDGF was used as a calibrator. Sandwich immunoassay was used for the measurement of human serum in this study.

### 4.8. HBMMSCs Recruitment Assay

The 24-well modified Boyden chambers with 8-μm pores-inserts (Corning Incorporated Life Sciences, Tewksbury, MA, USA) were used in HBMMSCs recruitment motility assay. The effect of HDGF, HDGF neutralizing antibody (HDGF-NAb) and isotype IgG on HBMMSCs motility was measured. HBMMSCs were placed in the upper chamber in the co-culture system filled with serum- and phenol red-free medium. The invasive cells on the bottom of insert were fixed with 4% paraforrmaldehyde and stained with 1μg/mL 4′-6-diamidine-2-phenylindole dihydrochloride (DAPI; Roche Molecular Biochemicals) for 30 min. The phenotypes of cell motility were counted with fluorescence microscopy at 40× and 100× magnifications, respectively.

### 4.9. Invasive Motility Assay

Human gastric cancer cell invasive motility assay was performed in the 24-well modified Boyden chambers containing inserts with 8-μm pores (Corning Incorporated Life Sciences). Inserts were pre-coated with collagen I (Sigma Chemical Co,) for detecting invasive motility of human gastric cancer cells. To measure the effect of HBMMSCs on invasive motility of gastric cancer cells, HBMMSCs were seeded in the lower compartment, and human gastric cells were placed in the upper chamber. The co-culture system was filled with serum- and phenol red-free medium and incubated for various periods, respectively. Invasive cells on the bottom of membrane filter were fixed with 4% paraformaldehyde and stained with 1 μg/mL DAPI (Roche Molecular Biochemicals) for 30 min to detect cell nucleus (blue stains) by fluorescence microscopy at 40× and 100× magnifications, respectively.

### 4.10. Cell Viability Assay

To determine the effect of HBMMSCs on gastric cancer cell viability, AGS cells (2.5 × 10^4^) were co-cultured with HBMMSCs (1 × 10^4^) for 24, 48, and 72 h and were subjected to 3-(4,5-dimethylthiazol-yl)-2,5-diphenyltetrazolium bromide (MTT; Sigma Chemical Co.) assay. Human gastric cancer cells were seeded in the lower compartment, and HBMMSCs were placed in the upper insert with 0.45-μm pores (Corning Incorporated Life Sciences) for co-culture. The upper inserts with HBMMSCs then were removed to allow only detecting the survival of human gastric cancer cells in the lower compartment. The absorbance of blue formazan crystals was measured at 570 nm in an enzyme-linked immunosorbent assay (ELISA) machine. The cell viability was measured according to the absorbance corrected to a background reading.

### 4.11. Multilineage Differentiation

#### 4.11.1. Osteogenic Differentiation

HBMMSCs (P4 to P7) were cultured in the osteogenic medium for 3 weeks for osteogenic differentiation induction. Culture medium was changed twice weekly. Osteogenesis was measured at weekly intervals. Osteogenic differentiation medium consists of IMDM containing 10 mM β-glycerol phosphate (Sigma Chemical Co.), 0.1 μM dexamethasone (Sigma Chemical Co.), and 50 μM L-Ascorbate-2 phosphate (Sigma Chemical Co.).

#### 4.11.2. Adipogenic Differentiation

HBMMSCs (P4 to P7) were cultured in the adipogenic differentiation medium for 3 weeks for adipogenic differentiation induction. Culture medium was changed twice weekly. Adipogenesis was measured at weekly intervals. Adipogenic differentiation medium consists of IMDM containing 0.5 mM 3-isobutyl-1-methylxanthine (Sigma Chemical Co.), 1 μM hydrocortisone (Sigma Chemical Co.), 0.1 mM indomethacin (Sigma Chemical Co.), and 10% horse serum (Sigma Chemical Co.).

#### 4.11.3. Chondrogenic Differentiation

HBMMSCs (P4 to P7) were transferred into 15-mL polypropylene tube and centrifuged at 1000 rpm for 5 min to form a pelleted micromass at the bottom of the tube and then treated with chondrogenic induction medium for 3 weeks. Chondrogenic differentiation medium was changed twice weekly. Chondrogenesis was measured at weekly intervals. Chondrogenic differentiation medium consists of high-glucose DMEM (Bio-fluid, Rockville, MD, USA) containing 0.1 μM dexamethasone, 50 μg/mL AsA, 100 μg/mL sodium pyruvate (Sigma Chemical Co.), 40 μg/mL proline (Sigma Chemical Co.), 10 ng/mL TGF-β1, and 50 mg/mL ITS+ premix (6.25 μg/mL insulin, 6.25 μg/mL transferrin, 6.25 ng/mL selenius acid, 1.25 mg/mL bovine serum albumin (BSA), and 5.35 mg/mL linoleic acid (Becton Dickinson, Franklin Lakes, NJ, USA)).

#### 4.11.4. Cytochemical Staining

For analysis of mineralized matrix, cells were fixed with 4% formaldehyde and followed by von Kossa staining for calcium, using 1% silver nitrate (Sigma Chemical Co.) under UV light for 45 min, 3% sodium thiosulfate (Sigma Chemical Co.) for 5 min, and then counterstained with van Gieson (Sigma Chemical Co.) for 5 min. For oil-red O staining, cells were fixed with 4% formaldehyde, stained with oil-red O (Sigma Chemical Co.) for 10 min, and then counterstained with Mayer hematoxylin (Sigma Chemical Co.) for 1 min. Alcian blue staining (Sigma Chemical Co.) was used for detection of chondrogenic differentiation. After incubate in the dark for 45 min at room temperature, cartilage was stained intense dark blue, whereas other tissues were mostly stained light blue.

#### 4.11.5. Histological Analysis

Chondrogenic differentiation was identified after pellets were fixed in 4% formaldehyde, dehydrated in serial dilutions of ethanol, and embedded in paraffin blocks. Blocks then were into serial 4-μm sections and stained with H&E (Sigma).

### 4.12. Total RNA Extraction

Cells were homogenized in RNA lysis/binding buffer (The High Pure RNA Tissue Kit (Roche Applied Science, Mannheim, Germany)) for isolation of total RNA.

### 4.13. Reverse Transcription and Real-Time PCR Assay

The standard protocols of reverse transcription and real-time PCR protocol were used in this study. The samples were incubated at 25 °C for 10 min in the step of reverse transcription. A hot start (10 min at 95 °C, 1 cycle) was initiated in the step of Real-time PCR, the samples were then subjected to 40 reaction cycles (95 °C for 15 s and 60 °C for 1 min). Data was analyzed by StepOne real-time PCR system (Applied Biosystems, Foster City, CA, USA). Primers used for target gene expression (MISSION BIOTECH, Taipei, Taiwan) are listed in Table 1.

### 4.14. Statistical Analysis

Each experiment was repeated at least three times. Results were presented as the mean ± SD, and statistical differences were analyzed using the Student’s t test. Significance was defined at the *p* < 0.05 or 0.01 levels.

## 5. Conclusions

Gastric cancer is one of the most common cancers worldwide, and is also the second leading cause of cancer-related mortality. Accumulating studies suggest that BMMSCs might contribute to t cancer progress through generation of myofibroblast/carcinoma-associated fibroblast (CAFs). In this study, we demonstrated that HDGF recruits HBMMSCs, and stimulates the differentiation of CAFs from HBMMSCs. HBMMSCs upregulate the capacity of cell survival and invasive motility of human gastric cancer cells. These findings suggest that HDGF might play a critical role in gastric cancer progress.

## Figures and Tables

**Table 1:** Markers for myofibroblasts and fibroblasts.

Primer		Sequence (5′ to 3′)	Orientation
**Myofibroblast**	human α smooth muscle actin (α-SMA)	ACCCACAATGTCCCCATCTA	Forward
	human α smooth muscle actin (α-SMA)	GAAGGAATAGCCACGCTCAG	Reverse
	human procollagen α-I	GTGCTAAAGGTGCCAATGGT	Forward
	human procollagen α-I	ACCAGGTTCACCGCTGTTAC	Reverse
	human tropomyosin 1	GAAGCTCGACAAGGAGAACG	Forward
	human tropomyosin 1	TTTTGCAGTGACACCAGCTC	Reverse
	human desmin	CAAGCTGCAGGAGGAGATTC	Forward
	human desmin	GAGATTCAATTCTGCGCTCC	Reverse
	human fibroblast activation protein α (FAP-α)	CGATACCACTTACCCTGCGT	Forward
	human fibroblast activation protein α (FAP-α)	ATCAGTAACCCACGTGAGCC	Reverse
**Fibroblast**	human prolyl 4-hydroxylase A1 (PHA1)	GGCAGCCAAAGCTCTGTTAC	Forward
	human prolyl 4-hydroxylase A1 (PHA1)	AAAGCAGTCCTCAGCCGTTA	Reverse
	human fibroblast specific protein-1 (FSP-1)	GATGAGCAACTTGGACAGCA	Forward
	human fibroblast specific protein-1 (FSP-1)	CTTCCTGGGCTGCTTATCTG	Reverse

Oligonucleotide primers used for q-PCR.

## Supplementary Materials

The following are available online at http://www.mdpi.com/2072-6694/10/12/479/s1, Figure S1, Figure S2, Figure S3, Figure S4 and Figure S5.
