# Supplementary material for: Helicobacter pylori Infection-Induced Hepatoma-Derived Growth Factor Regulates the Differentiation of Human Mesenchymal Stem Cells to Myofibroblast-Like Cells
Source: Cancers (Basel). 2018 Nov 30;10(12):479. doi: 10.3390/cancers10120479 (PMC6316704; doi:10.3390/cancers10120479)
Supplement: Supplementary file 1 [file cancers-10-00479-s001.pdf]

cancers

Supplemental Materials: *Helicobacter pylori* Infection-Induced Hepatoma-Derived Growth Factor Regulates the Differentiation of Human Mesenchymal Stem Cells to Myofibroblast-Like Cells

Chung-Jung Liu, Yao-Kuang Wang, Fu-Chen Kuo, Wen-Hung Hsu, Fang-Jung Yu, Shuchen Hsieh, Ming-Hong Tai, Deng-Chyang Wu and Chao-Hung Kuo

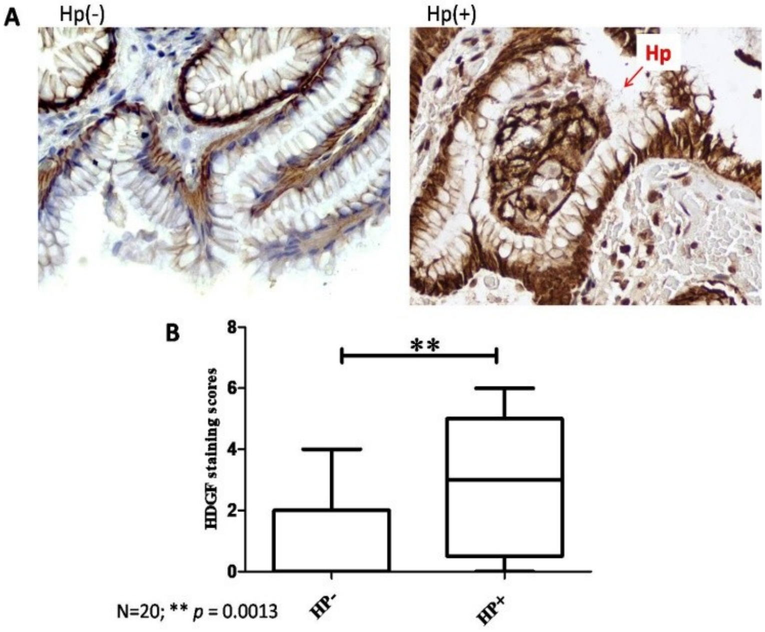

Figure S1. We analyzed HDGF protein expression in the human gastric tissue with or without HP infection (HP-, *N* = 20 cases; HP+, *N* = 20 cases), using IHC staining. We found that HDGF is significantly expressed in HP-infected gastric tissue ($p = 0.0013$).

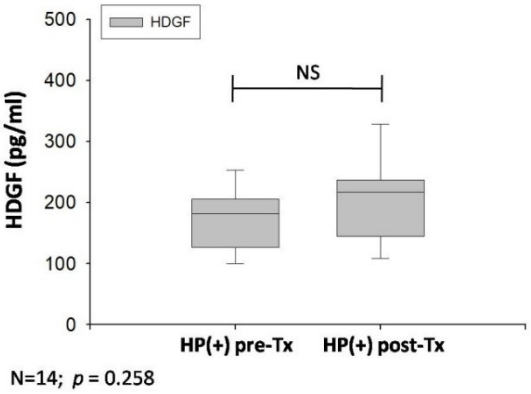

Figure S2. We found serum HDGF is not significantly changed when compare HP-infected (HP+) patients before and after HP eradication (pre-TX and post-TX). We speculated that HP infection has induced the changes of many host's genes and proteins by virulence factors of HP, even though HP is eradicated.

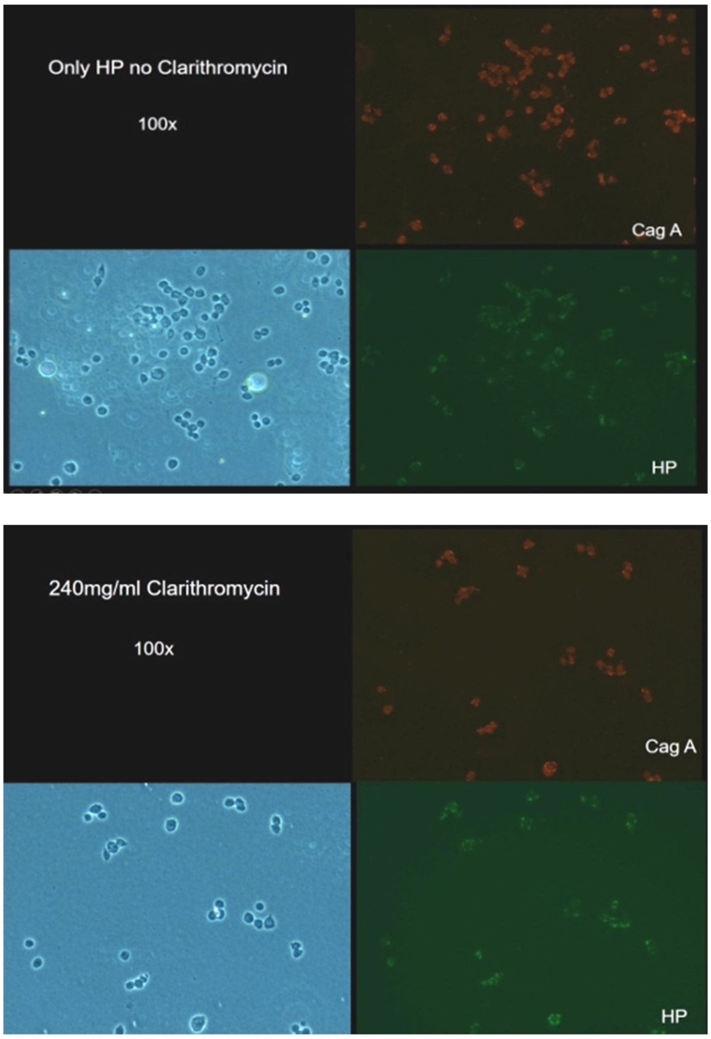

Figure S3. We have established the HP49503 infection in the AGS cells culture. We observed the adhesion of HP49503 in the presence and absence of Clarithromycin (HP eradication), using immunefluorescence antibodies against HP and CagA. We found that HP49503 possesses the strong adhesion ability even though HP is eradicated (sterilized/killed).

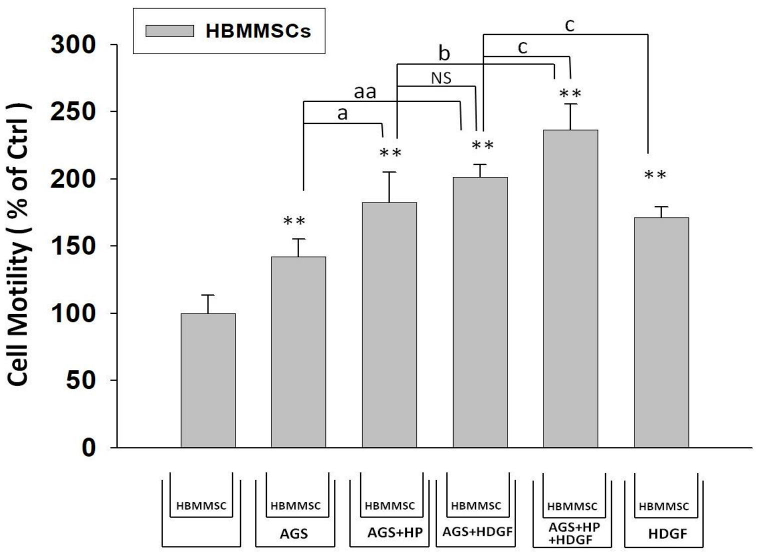

Figure S4. We have examined the chemotactic potential of AGS ± Hp in the presence or absence of HDGF. The results suggested that HP infection and/or HDGF treatment enhance the capacity of HBMMSCs recruitment.

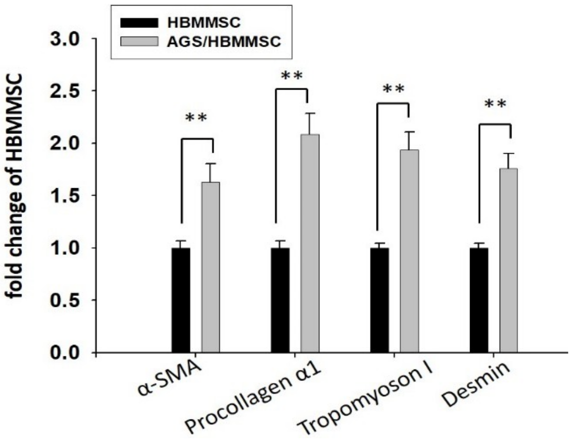

Figure S5. We examined whether AGS cells could trigger the differentiation of HBMMSCs in the transwell co-culture system for 48 hours. The results suggested that AGS cells might contribute to the differentiation of the HBMMSCs into myofibroblast-like cells.

© 2018 by the authors. Licensee MDPI, Basel, Switzerland. This article is an open access article distributed under the terms and conditions of the Creative Commons Attribution (CC BY) license (http://creativecommons.org/licenses/by/4.0/).
